# Prenatal caffeine exposure induces a poor quality of articular cartilage in male adult offspring rats *via* cholesterol accumulation in cartilage

**DOI:** 10.1038/srep17746

**Published:** 2015-12-07

**Authors:** Hanwen Luo, Jing Li, Hong Cao, Yang Tan, Jacques Magdalou, Liaobin Chen, Hui Wang

**Affiliations:** 1 Department of Orthopedic Surgery, Zhongnan Hospital of Wuhan University, Wuhan 430071, China; 2 Department of Pharmacology, Basic Medical School of Wuhan University, Wuhan 430071, China; 3 UMR 7561 CNRS-Universitéde Lorraine, Faculté de Médicine, Vandoeuvre-lès-Nancy, France; 4 Hubei Provincial Key Laboratory of Developmentally Originated Disease, Wuhan 430071, China

## Abstract

Epidemiological investigations indicate that osteoarthritis is associated with intrauterine growth retardation (IUGR) and abnormal cholesterol metabolism. Our previous studies showed that prenatal caffeine exposure (PCE) induced chondrogenesis retardation in IUGR offspring rats. The current study sought to investigate the effects of PCE on male IUGR offspring rats’ articular cartilage, and the mechanisms associated with abnormal cholesterol metabolism. Based on the results from both male fetal and adult fed a high-fat diet (HFD) studies of rats that experienced PCE (120 mg/kg.d), the results showed a poor quality of articular cartilage and cholesterol accumulation in the adult PCE group. Meanwhile, the serum total cholesterol and low-density lipoprotein-cholesterol concentrations were increased in adult PCE offspring. We also observed lower expression of insulin-like growth factor1 (IGF1) and impaired cholesterol efflux in adult articular cartilage. Furthermore, the expression of cartilage functional genes, components of the IGF1 signaling pathway and cholesterol efflux pathway related genes were decreased in PCE fetal cartilage. In conclusion, PCE induced a poor quality of articular cartilage in male adult offspring fed a HFD. This finding was shown to be due to cholesterol accumulation in the cartilage, which may have resulted from intrauterine reduced activity of the IGF1 signaling pathway.

Osteoarthritis (OA) is a chronic joint disease characterized by the major pathological feature of articular cartilage degeneration. OA is the most common cause of joint pain in the elderly^1^. The relationship between metabolic syndrome (MS) and OA has been highlighted in previous etiological studies of OA^2^, and more and more researchers agree that OA should be characterized as a metabolic disorder^3^,^4^. Based on a large amount of epidemiological evidence, Barker showed that the incidence of adult MS in fetuses with intrauterine growth retardation (IUGR) was higher than that in normal fetuses, which led to the theory of “the intrauterine origin of adult disease”^5^,^6^. Epidemiological data have further showed that hand OA is significantly associated with lower birth weight in males, and females show a similar trend^7^. An analogous conclusion was also found in another epidemiological study that focused on the relationship between low birth weight and lumbar spine OA^8^. Together, these reports indicate that changes to cartilage during intrauterine development may increase susceptibility to OA^9^.

Many studies have confirmed a poor quality of cartilage in OA patients, and cartilage quality is significantly associated with the onset of OA^10^,^11^. Cellular cholesterol accumulation can induce cytotoxicity and cellular damage, but can be prevented through the cholesterol efflux system^12^,^13^. Similar to atherosclerosis, the inhibition of the cholesterol efflux system in OA chondrocytes, characterized as lower liver X receptor (LXR) expression, results in the cellular accumulation of cholesterol^14^. Epidemiological data^15^,^16^ have further showed that OA is significantly associated with cardiovascular disease and the blood cholesterol level^17^. Hence, the underlying mechanisms of fetal-origin OA may include cholesterol accumulation in cartilage, which maybe induced by hypercholesteremia.

Caffeine is a methylxanthine alkaloid that is widely present in coffee, tea, soft drinks, food and some drugs. Many studies have revealed that caffeine ingestion during pregnancy correlates with IUGR. Our previous studies showed that prenatal caffeine exposure (PCE) could elevate the maternal serum glucocorticoid (GC) concentration and over-expose the fetus to maternal GC^18^,^19^,^20^, thus resulting in retardation of chondrogenesis by down-regulation of insulin-like growth factor1 (IGF1) signaling pathway in fetal growth plate cartilage^21^. IGF1 is a key factor for cartilage anabolism and maintaining the cartilage phenotype^22^. The expression and secretion of IGF1 can be decreased by GC^23^, and IGF1 may take part in the regulation of cholesterol metabolism^24^,^25^. However, no studies have addressed whether PCE induces a poor quality of cartilage in adult offspring and whether this poor quality cartilage may be derived from cholesterol accumulation and changes in intrauterine metabolic programming.

A high-fat diet (HFD) is one of the main environmental factors accounting for the incidence of MS^26^, particularly in environments where HFD are prevalent. In the present study, a rat IUGR model was established by PCE, as described in our previous studies^19^,^27^. A post-weaning HFD was given to induce MS and hypercholesteremia in offspring as an induction factor. We then observed the changes in cartilage quality in adult IUGR offspring. Furthermore, we investigated the fetal origin mechanisms related to both cholesterol influx (hypercholesteremia) and efflux in articular cartilage. This study should clarify the etiopathogenesis of adult OA and provide evidence for the early prevention and treatment of fetal-origin OA.

## Results

### Body weight and its rate of gain in adult male offspring with a post-weaning HFD

**Figure 1:**
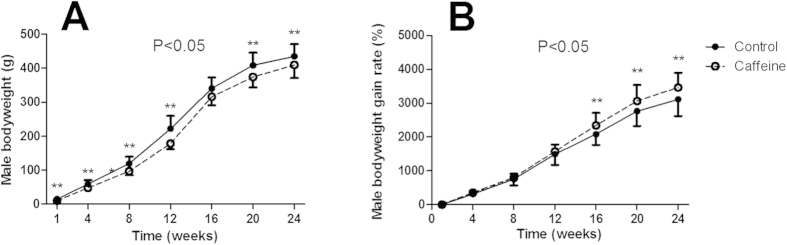
Changes in body weight, weight gain rate in prenatal caffeine exposure (PCE) male offspring fed post-weaning high-fat diet. (**A**) bodyweight; (**B**) bodyweight gain rate. Mean ± S.E.M., n = 8. ^**^P < 0.01 vs. control of each time point.

As shown in Fig. 1A, the weight loss of male PCE offspring was more than that of their control counterparts in postnatal week (PW) 1 (*P* < 0.01). When fed a post-weaning HFD, the body weight of male PCE offspring remained significantly decreased in PW4-PW24 (Fig. 1A, *P* < 0.01). However, the weight growth rate turned out to be increased from PW16 to PW24 (Fig. 1B, *P* < 0.01). Through the analysis of repeated measures, the results showed that the body weights of the PCE group were lower than those of the control group (*P* < 0.05). However, the PCE group gain rate was higher than that of the control group (Fig. 1A,B, *P* < 0.05).

### Histological observations and concentration of total cholesterol (TCH) in articular cartilage of adult male offspring

**Figure 2:**
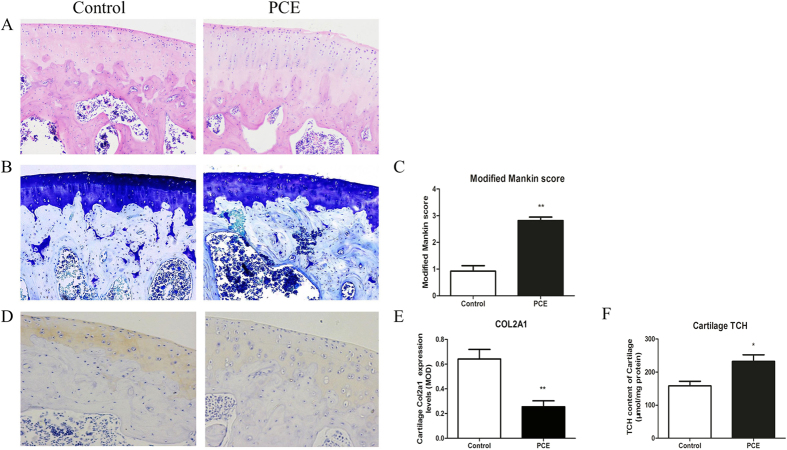
Changes in histology and total cholesterol (TCH) content in articular cartilage of prenatal caffeine exposure (PCE) male offspring fed post-weaning high-fat diet. (**A**) haematoxylin-eosin staining (200×); (**B**) toluidine blue staining (200×); (**C**) modified Mankin’s scores; (**D**) immunohistochemical staining ofα1 chain of type II collagen gene (Col2α1); (**E**) mean optical density (MOD) of Col2α1; (**F**) TCH content. Mean ± S.E.M., n = 5 offspring from 8 pregnant rats. ^*^P < 0.05, ^**^P < 0.01 vs. control.

Hematoxylin and eosin (HE) staining was performed to examine the femoral articular cartilage (Fig. 2A). The cartilage in control rats showed a normal chondrocytes structure, an intact surface, and ordered layers. In comparison, cartilage from the PCE group showed an irregular surface with roughing and derangement of the cells in the tangential zone. As shown by the toluidine blue staining in Fig. 2B, in the control group, typical staining of matrix and a tidemark were observed. In the PCE group, matrix staining was reduced and uneven, and the tidemark had disappeared. By employing a modified Mankin’s score, we found that PCE significantly increased the score for cartilage (*P* < 0.01). We used the modified Mankin’s score because it was found to be more sensitive than the Osteoarthritis Research Society International (OARSI) scoring system when evaluating the early impairment of cartilage^28^. Immunohistochemical staining was performed to examine the α1 chain of type II collagen (Col2α1) (Fig. 2D,E), the mean optical density (MOD) of which was lower in the PCE group (*P* < 0.01). Moreover, the concentration of TCH in the cartilage was significantly increased in the PCE group compared to the control group (Fig. 2F, *P* < 0.05). These results showed cholesterol accumulation and a poor quality of articular cartilage in adult offspring of the PCE group.

### Serum cholesterol phenotypes of male adult offspring

**Figure 3:**
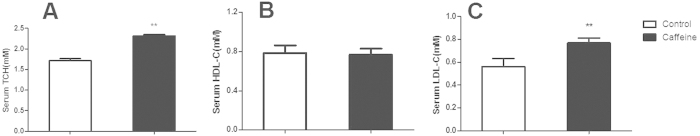
Changes in serum cholesterol concentrations in prenatal caffeine exposure (PCE) male offspring fed post-weaning high-fat diet. (**A**): total cholesterol concentration (TCH); (**B**): high-density lipoprotein-cholesterol (HDL-C) concentration; (**C**): low-density lipoprotein-cholesterol (LDL-C) concentration. Mean ± S.E.M., n = 8. ^**^P < 0.01 vs. control.

As shown in Fig. 3A–C, the concentrations of serum TCH and low-density lipoprotein-cholesterol (LDL-C) in the PCE group were significantly increased when compared with the control group (Fig. 3A,C, *P* < 0.01). The concentration of serum high-density lipoprotein-cholesterol (HDL-C) in the PCE group was nearly the same as that in the control group.

### IGF-1 and cholesterol efflux pathway -related protein expression in male adult articular cartilage

**Figure 4:**
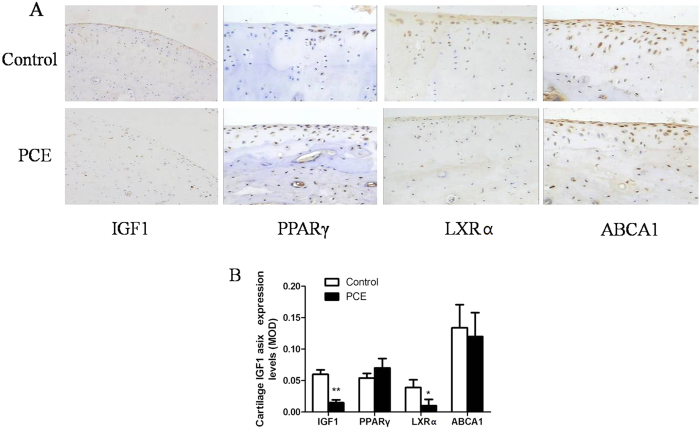
Changes in insulin like growth factor 1 (IGF1) and cholesterol efflux pathway-related protein expression of articular cartilage in prenatal caffeine exposure (PCE) male offspring fed post-weaning high-fat diet. (**A**): typical immunohistochemistry (200×); (**B**): mean optical density (MOD). PPARγ, peroxisome proliferator-activated receptor γ; LXRα, liver X receptor α; ABCA1, ATP-binding cassette transporter A1. Mean ± S.E.M., n = 5 offspring from 8 pregnant rats. ^*^P < 0.05, ^**^P < 0.01 vs. control.

Immunohistochemistry analysis (Fig. 4A,B) showed that the protein expression of IGF-1 in the PCE group was significantly reduced when compared with the control group (*P* < 0.01). In addition, cholesterol efflux-related LXRα protein expression in the PCE group was also lower than that in the control group (*P* < 0.05). However, the protein expression levels of peroxisome proliferator-activated receptor-γ (PPARγ) and ATP-binding cassette transporter A1 (ABCA1) were not significantly altered in the PCE group.

### The expression of IGF1 and cholesterol efflux in male fetal articular cartilage

**Figure 5:**
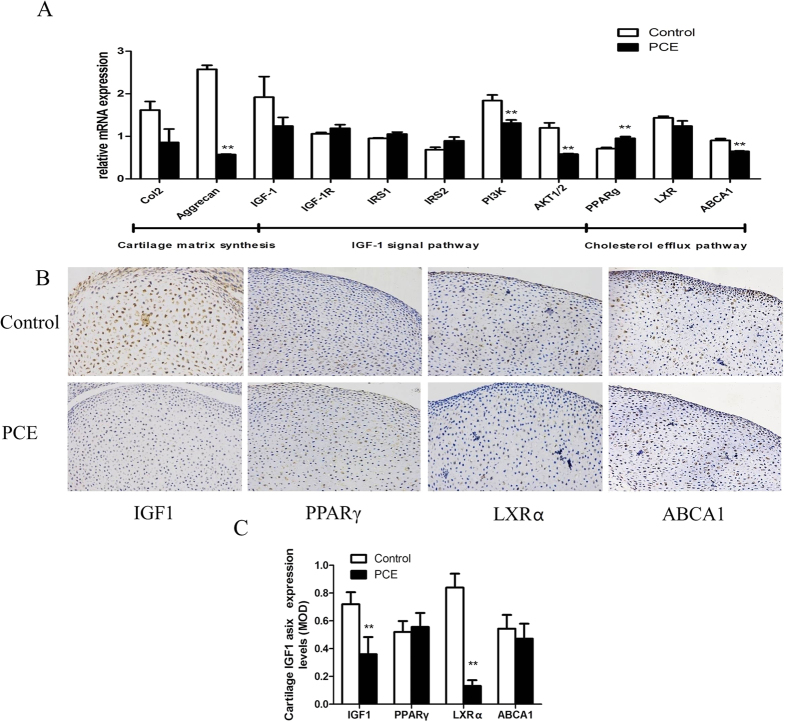
Changes in insulin-like growth factor 1 (IGF1) and cholesterol efflux pathway expression in prenatal caffeine exposure (PCE) male fetal articular cartilage. (**A**): Multiplex genes expression of fetal articular cartilage; (**B**): typical immunohistochemistry (200×); (**C**): mean optical density (MOD). IGF-1R, insulin-like growth factor 1 receptor; IRS1, insulin receptor substrate 1; IRS2, insulin receptor substrate 2; PI3K, phosphatidylinositol 3-kinase; AKT1/2, serine–threonine protein kinase 1/2; Col2α1, α1 chain of type II collagen gene; PPARγ, peroxisome proliferator-activated receptor γ; LXRα, liver X receptor α; ABCA1, ATP-binding cassette transporter A1. Mean ± S.E.M., n = 5 offspring from 8 pregnant rats. ^**^P < 0.01 vs. control.

Alterations in fetal articular cartilage multiplex gene expression between the control and PCE groups are shown in Fig. 5A. The detected genes were divided into three categories: cartilage functional genes, IGF-1 signaling pathway-related genes and cholesterol efflux pathway-related genes. Compared with` the control, the mRNA expression of *Aggrecan* and *Col2α1*, which are genes that reflect cartilage function, were significantly decreased (P < 0.01) or exhibited a decreasing trend (*P* < 0.1). Moreover, a downswing of the IGF1 (*P* < 0.1) and a significant decrease in *Pi3k*, *Akt1/2* (*P* < 0.01) mRNA expression were observed. Furthermore, an increase in the cholesterol efflux pathway-related gene, *Pparγ* was found (*P* < 0.01), whereas the downstream gene *Abca1* was decreased remarkably (*P* < 0.01). As shown in Fig. 5B, immunohistochemistry analysis demonstrated that the protein expression of IGF1 and LXRα in the PCE group was significantly reduced compared to the control group (*P* < 0.05, *P* < 0.01), while PPARγ and ABCA1 protein expression was not significantly altered in the PCE group.

## Discussion

Human epidemiological evidence indicates that caffeine intake during pregnancy is associated with human IUGR^29^. Furthermore, some studies have shown that caffeine intake in some pregnant women is >300 mg/d (>4.3 mg/kg.d)^30^,^31^, which is associated with an increased risk for small for gestational age according to the World Health Organization (WHO)^32^. Using the dose conversion between humans and rats (human:rat 1:6.17 by body surface area comparisons)^33^, the dose of 120 mg/kg.d used in the present study is comparable to a dose of 19.5 mg/kg.d in humans. In our previous studies^18^, we used 20, 60 and 180 mg/kg.d of caffeine, and observed some multi-index changes at 20 mg/kg.d (which is equivalent to 1.5–2.2 cups of coffee based on a standard cup of coffee that contains 100 mg to 150 mg of caffeine on average) and a clear dose-effect relationship. Furthermore, after an intake of 120 mg/kg.d caffeine, we found that the caffeine concentrations were 254 ± 11 μM (49 ± 2 μg/ml) and 155 ± 28 μM (29 ± 5 μg/ml) in maternal and fetal blood, respectively^34^, which did not reach the clinical dose of intoxication (~80 μg/ml)^35^. Taken together, the dose of 120 mg/kg.d caffeine was used in the present study, to achieve a stable IUGR model and explore the potential mechanism of fetal-origin OA.

Articular cartilage function is dependent on the molecular composition of its extracellular matrix (ECM), which consists mainly of proteoglycans and collagens^36^. The ECM also plays a very important role in maintaining the physiological function of articular cartilage. In the degenerative processes of OA, decreased levels of proteoglycans and collagens are typical observed^37^. In the present study, the articular cartilage showed evidence of intrauterine injury, which manifested as a disorderly structure of chondrocytes and ECM loss with a significantly increased modified Mankin’s score in PCE male adult offspring fed a HFD. We also found that the Col2α1 content in cartilage was decreased in the PCE group. These results suggest that PCE induces a poor quality of articular cartilage in male adult offspring fed a HFD, which could further result in increased susceptibility to OA.

Chondrocytes have a synthetic and secretory function and are vital in maintaining the stability of the ECM. However, cholesterol accumulation in chondrocytes will affect ECM synthesis and secretion, which will lead to a poor quality of articular cartilage^38^,^39^. It has been shown that cholesterol specifically accumulates in the superficial area of OA cartilage^40^. Moreover, the reduced expression of LXRs and ApoA1 has been described in human OA cartilage, leading to impaired cholesterol efflux and intracellular lipid deposition in OA chondrocytes^39^. Animal experiments have also shown that ApoA1^−/−^ mice spontaneously develop OA when fed a Western-type diet due to alterations in HDL metabolism^41^. Furthermore, exogenous cholesterol was shown to reduce Col2α1 mRNA expression in human chondrocytes^38^. In this study, we found that the TCH level of cartilage was significantly enhanced in PCE male adult offspring rats fed a HFD. Therefore, we conclude that the poor quality of articular cartilage in the PCE offspring was associated with local cholesterol accumulation.

GC are key metabolic hormones regulating fetal growth, development and maturity *in utero*, while IGF1 plays an insulin-like growth-promoting role and is the main factor associated with IUGR and postnatal catch-up growth^42^,^43^. It has been reported that GC can inhibit IGF1 expression^23^, and Fowden reported that serum IGF1 levels could be modified by changes in fetal serum GC concentrations induced by adverse intrauterine conditions^44^. IUGR offspring typically exhibit a rapid catch-up growth period, accompanied by an increased serum IGF1 level after birth^45^. PCE can be considered a pregnancy stress in the current study, it could affect offspring health by elevating the maternal serum GC concentration^18^,^19^,^20^. Our previous studies demonstrated that IUGR fetuses induced by PCE showed enhanced GC and decreased IGF1 levels in the serum^21^,^27^. However, the serum IGF1 concentration was increased and catch-up growth occurred after birth^19^,^34^. In the present study, we also observed a catch-up growth in the PCE male offspring fed a HFD.

Several studies have demonstrated that catch-up growth is closely related to an increased risk of developing adult MS^45^,^46^,^47^, which can be represented as an increased level of serum cholesterol. Some epidemiological surveys have shown an association between hypercholesterolemia and OA^48^,^49^. Serum cholesterol can affect the content of cholesterol in cartilage *via* synovial fluid and subchondral bone^50^. In this study, the results showed that serum TCH and LDL-C levels were increased in the PCE male offspring fed a HFD, which was due to altered intrauterine programming in the fetal liver caused by over-exposure to maternal GC (unpublished data). These results indicate that hypercholesterolemia induced by PCE is one of the main reasons for cholesterol accumulation in cartilage.

Adverse external factors during embryonic or childhood development may have lifelong consequences, resulting in tissue and/or organ functional or gene expressional changes during each developmental stage. These changes are usually maintained from puberty to adulthood and can lead to a series of adverse effects^51^. Epigenetic modifications might play a role in IUGR programmed metabolic dysfunction^52^. It is generally known that IGF1 is an important mediator of cartilage growth^22^. Our previous study showed that PCE induces skeletal growth retardation by inhibiting IGF1 expression in the liver and growth plate cartilage, which is mediated by epigenetic modification^21^. In the present study, the results showed that the IGF1 pathway was suppressed in IUGR fetal articular cartilage, and these alterations could be maintained to adulthood. These findings suggest that PCE may lead to impaired activity of the articular cartilage IGF1 pathway in IUGR fetuses.

IGF1, through the phosphorylation of PI3K/AKT promotes various downstream target genes by activating cascade reactions. In addition, the IGF1 signaling pathway may regulate the cholesterol efflux pathway, and PPARγ and LXRα can be activated by PI3K^24^,^25^. ABCA1 serves as a lipid pump that effluxes cholesterol and phospholipids from cells to Apolipoprotein A1, and this process is regulated by PPARγ and LXRα^53^,^54^. Several studies have revealed that osteoarthritic chondrocytes contain intracellular lipid deposits, and that the expression of cholesterol efflux genes, such as LXRα, is dramatically reduced in OA articular cartilage samples^39^,^55^. In addition, Tsezou found that treatment of OA chondrocytes with the LXR agonist TO-901317 significantly increased ABCA1 expression levels, as well as decreased cholesterol efflux lipid deposits within the OA chondrocytes^39^. Therefore, altered cholesterol efflux could be a risk factor and/or consequence of OA^40^. In the present study, the results showed that the IGF1/PI3K/AKT signaling pathway and LXRα/ABCA1 mRNA and/or protein expression were decreased in PCE male fetal articular cartilage, and these changes were partially extended to adulthood. This finding suggests that cholesterol accumulation in the PCE male offspring cartilage may have originated from altered cholesterol efflux due to low functional intrauterine programming of the IGF1 signaling pathway.

**Figure 6:**
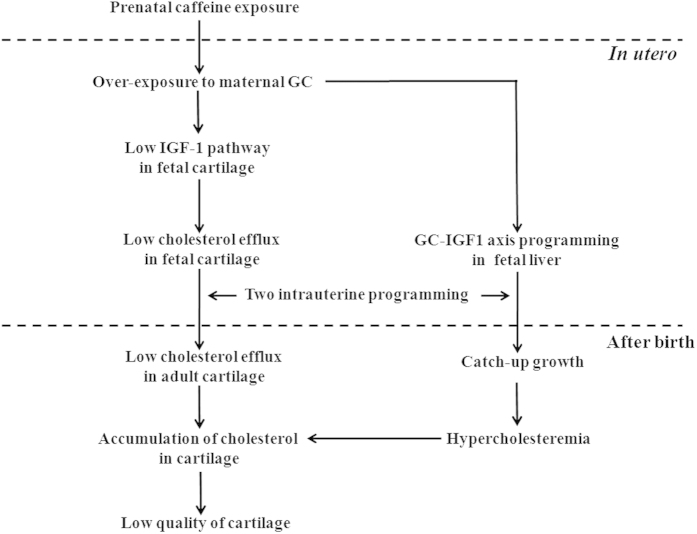
Prenatal caffeine exposure induces poor quality of articular cartilage in male adult offspring rats fed high-fat diet *via* the cholesterol accumulation in cartilage. IGF1, insulin like growth factor 1; GC, glucocorticoids.

In conclusion, PCE induced a poor quality of articular cartilage in male adult offspring rats fed HFD, which may suggest a “two-programming” mechanism (Fig. 6). On the one hand, the intrauterine liver GC-IGF1 axis programming induced postnatal catch-up growth and hypercholesterolemia, which increased the cholesterol influx in chondrocytes. On the other hand, the altered cholesterol efflux in chondrocytes induced by low functional intrauterine programming of the IGF1 signaling pathway, resulting in a decreased outlet of cholesterol. Both of these “two programming” jointly induced cholesterol accumulation and a poor quality of the articular cartilage, thus increase the susceptibility to OA. This study provides a valuable experimental basis for explaining the fetal-origin OA.

## Materials and Methods

### Materials

Caffeine (CAS #58-08-2, >99% purity) was purchased from Sigma-Aldrich Co., Ltd. (St Louis, MO, USA). Isoflurane was purchased from Baxter Healthcare Co. (Deerfield, IL, USA). TCH, LDL-C and HDL-C assay kits were purchased from Sangon Biotech Co., Ltd. (Shanghai, China). Tissue TCH assay kits were purchased from Applygen Tech, Inc. (Beijing, China). Reverse transcription and quantitative PCR (Q-PCR) kits were purchased from Takara Biotechnology Co., Ltd. (Dalian, China). GeXP multiplex gene expression analysis kits were purchased from Beckman-Coulter Inc. (Fullerton, CA, USA). The oligonucleotide primers for rat Q-PCR genes (PAGE purification) and GeXP multiplex gene expression analysis (HPLC purification) were synthesized by Sangon Biotech Co., Ltd. (Shanghai, China). Polyclonal antibodies for Col2α1, IGF1, PPARγ, LXRα and ABCA1 were obtained from SantaCruz Biotechnology, Inc. (Santa Cruz, CA, USA). Other chemicals and agents were of analytical grade.

### Animals and treatment

Animal experiments were performed in the Center for Animal Experiment of Wuhan University (Wuhan, China), which has been accredited by the Association for Assessment and Accreditation of Laboratory Animal Care International (AAALAC International). The protocol was approved by the Committee on the Ethics of Animal Experiments at the Wuhan University School of Medicine (Permit Number: 14016). All animal experimental procedures were performed in accordance with the Guidelines for the Care and Use of Laboratory Animals (eighth edition) by the National Research Council of the United States National Academies.

Specific pathogen-free (SPF) Wistar rats, including females weighing 200–240 g and males weighing 260–300 g, were obtained from the Experimental Center of Hubei Medical Scientific Academy (No. 2009-0004, Hubei, China). Animals were housed in metal cages with wire-mesh floors in an air-conditioned room under standard conditions (room temperature: 18–22 °C; humidity: 40%–60%; light cycle: 12 h light-dark cycle; 10–15 air changes per hour) and allowed free access to rat chow and gap water. All rats were acclimated one week before experimentation, and then two female rats were placed together with one male rat overnight in a cage. Upon confirmation of mating by the appearance of sperm in a vaginal smear, the day was taken as gestational day (GD) 0. Pregnant females were transferred to individual cages at GD19. Pregnant rats were randomly divided into two groups: the control group and PCE group. Starting from GD11 until GD20, the PCE group was administrated caffeine (120 mg/kg.d) as described previously^18^, while the control group was given the same volume of distilled water. All pregnant rats were fed with lab chow. On GD20, 8 randomly selected pregnant rats with 10–14 live fetuses from each group were anesthetized with isoflurane and euthanized in a room separate from that the other pregnant rats were kept. The male fetuses were quickly removed and weighed and IUGR was diagnosed when the body weight of a fetus was two standard deviations less than the mean body weight of the fetuses in the control group. Fetal femurs were separated under a dissecting microscope and collected. The samples collected from each littermate were pooled together and immediately frozen in liquid nitrogen, followed by storage at -80°C for subsequent analyses. Fetal left femurs were randomly selected (one per litter) and fixed in phosphate-buffered 4% paraformaldehyde solution for 24 h before being decalcified in EDTA, dehydrated in alcohol and embedded in paraffin. The remaining femurs of the littermates were pooled for PCR analysis.

**Figure 7:**
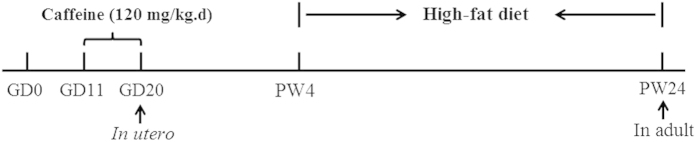
The schedule of animal treatment from gestation day (GD) 0 to postnatal week (PW) 24.

The other pregnant rats (n = 8 for each group) underwent normal delivery. On postnatal day 1 (PD1), the numbers of pups were normalized to 8 pups per litter to assure adequate and standardized nutrition until weaning PW4. After weaning, one male pup per litter was randomly selected and assigned to either the control group or the PCE group. All pups were weaned to a HFD before being sacrificed on PW24. The HFD was described previously^56^ and contains 88.0% corn flour, 11.5% lard, and 0.5% cholesterol, which provided 18.9% of the energy content as protein, 61.7% as carbohydrate, and 19.4% as fat. The bodyweights of the offspring rats were measured weekly until PW24, and the corresponding gain rates were calculated as follows: gain rate of bodyweight (%) = [(bodyweight at PWX–bodyweight at PW1)]/bodyweight at PW1 × 100^19^. At PW24, the offspring were anesthetized with isoflurane and decapitated in a room separate from that the other animals were kept. Serum was prepared and stored at −80 °C prior to analysis. Both femurs were dissected; the left femurs were fixed in a 4% paraformaldehyde solution for histological examination, and the right femurs were stored at −80 °C for cholesterol concentration detection. The schedule of animal treatment is shown in Fig. 7.

### Analysis for blood samples and cartilage

Serum TCH, LDL-C and HDL-C concentrations (biochemical assay) were detected by using assay kits according to the manufacturer’s protocol^57^. Cartilage TCH was measured with a tissue TCH biochemical assay kit.

### Histological and immunohistochemical assays

For histological analysis, femur sections of adult offspring were stained with HE and toluidine blue. The femurs were sectioned sagittally at a thickness of 5 μm. Sections were observed and photographed with an Olympus AH-2 light microscope (Olympus, Tokyo, Japan). Five sections from the femurs were randomly chosen to measure the quality of cartilage in each sample. Each section of adult femur was evaluated with the Mankin’s histological grading system in a blindfolded manner^58^. Immunohistochemical assays were performed to determine the expressional levels of Col2α1, IGF1, PPARγ, LXRαand ABCA1 proteins in the articular cartilage of the lower end of the femur. The primary antibodies were rabbit anti-rat polyclonal and the secondary antibody was a goat anti-rabbit IgG. The intensity of staining was determined by measuring the MOD in 5 different fields for each sample. All images were captured using an Olympus AH-2 light microscope (Olympus, Tokyo, Japan). Analysis of the stained images was conducted using Image Pro Plus software (version 6.1). For each animal the average score was used for statistical analysis.

### Multiplex gene expression analysis

Gene expression of fetal femur articular cartilage was detected by Multiplex gene expression analysis, which included 3 housekeeping and 12 target genes and was run on the GenomeLabGeXP Genetic Analysis System (Beckman-Coulter) using the GenomeLab^TM^eXpress Profiler software (Beckman-Coulter, Fullerton, CA)^59^. Multiplex optimization (*e.g.,* primer validation and attenuation) was completed according to the manufacturer’s instructions. Briefly, a primer pair was considered valid if only one PCR product of less than one nucleotide differed from its predicted size after being run on the GenomeLabGeXP Genetic Analysis System^59^. The list of genes and primer pairs are given in Table 1. To account for the different scale of expression of each gene, the proportion of each reverse primer in the multiplex reverse transcription reaction was adjusted to obtain similar peak signals for each gene. Reverse transcription was performed using 100 ng of RNA as a template according to the GenomeLabGeXP Genetic Analysis System protocol. Real-time PCR amplification was performed using a mix of the forward primers, and the resulting reactions were analyzed by capillary electrophoresis on the GenomeLabGeXP using the GeXP Start kit reagents. Relative RNA expression levels were calculated relative to a pooled RNA standard using the GeXP Quant Tool software and were normalized to the GAPDH, β-actin, and HPRT expression levels.

### Statistical analysis

SPSS 17 (SPSS Science Inc., Chicago, Illinois) was used for data analysis. Quantitative data were expressed as the mean ± S.E.M., and were evaluated with an independent samples *t*-test. Repeated measures were used to analyze bodyweight and bodyweight gain rate. The number of samples were n = 8 for body weight and serum phenotypes, and n = 5 for cartilage indexes. Statistical significance was defined as *P* < 0.05.

## Additional Information

**How to cite this article**: Luo, H. *et al.* Prenatal caffeine exposure induces a poor quality of articular cartilage in male adult offspring rats via cholesterol accumulation in cartilage. *Sci. Rep.* **5**, 17746; doi: 10.1038/srep17746 (2015).

## Figures and Tables

**Table 1:** Primer sequences of GeXP analysis gene.

Genes	Forward primer	Reverse primer
IGF1	AGGTGACACTATAGAATACCAGCTGTTTCCTGTCTACAG	GTACGACTCACTATAGGGAAGATGTGAAGACGACATGATGT
IGF1R	AGGTGACACTATAGAATACAAGACAGAAGTCTGCGGTG	GTACGACTCACTATAGGGACCGGGTCTGTGATATTGTAGG
IRS1	AGGTGACACTATAGAATAGGACCGTCAATAGCTTAACTGG	GTACGACTCACTATAGGGAGTCACAGTGCTTTCTTGTTGCT
IRS2	AGGTGACACTATAGAATATGTCCCATCACTTGAAAGAAG	GTACGACTCACTATAGGGACCTGCCTCTTGGTTCCTTATC
PI3K	AGGTGACACTATAGAATACGATGGAATTGGAACGAGTG	GTACGACTCACTATAGGGACCAGACTTTCAAGTCGTGCA
AKT1/2	AGGTGACACTATAGAATAATGAACGACGTAGCCATTGTG	GTACGACTCACTATAGGGAATGATGAAGGTGTTGGGCCT
Aggrecan	AGGTGACACTATAGAATACGGACTGAAGTTCTTGGAGG	GTACGACTCACTATAGGGAGGTTGAGGGATGCTCACACT
Col2α1	AGGTGACACTATAGAATATCAAGGAGAAGCTGGACAGAA	GTACGACTCACTATAGGGACCCAGGGTTGCCATTAGAAC
PPARγ	AGGTGACACTATAGAATAGGCGATCTTGACAGGAAAGA	GTACGACTCACTATAGGGAGAAACTGGCACCCTTGAAAA
LXR**α**	AGGTGACACTATAGAATACCATTTCCAGGGTAACGAAG	GTACGACTCACTATAGGGAAGACATAGTGGGTCACGAAGC
ABCA1	AGGTGACACTATAGAATACGTCCTTGTGTCCATCTGTG	GTACGACTCACTATAGGGAAAGGGCTAGAACAGGCAGGT
β-actin	AGGTGACACTATAGAATAGTCCACCCGCGAGTACAAC	GTACGACTCACTATAGGGACCCACGTAGGAGTCCTTCTG
GAPDH	AGGTGACACTATAGAATATCTCTGCTCCTCCCTGTTCTAG	GTACGACTCACTATAGGGAGGTCAATGAAGGGGTCGTTG
Tublin	AGGTGACACTATAGAATATGTAAGAAGCAACACCTCCTC	GTACGACTCACTATAGGGACAGTGCGAACTTCATCAATAAC

IGF1, insulin-like growth factor 1; IGF1R, insulin-like growth factor 1 receptor; IRS1, insulin receptor substrate 1; IRS2, insulin receptor substrate 2; PI3K, phosphatidylinositol 3-kinase; AKT1/2, serine–threonine protein kinase 1/2; Col2α1, α1 chain of type II collagen gene; PPARγ: peroxisome proliferator-activated receptor γ; LXRα, liver X receptor α; ABCA1, ATP-binding cassette transporter A1; GAPDH, glyceraldehyde 3-phosphate dehydrogenase.
